# Invited Editorial. COVID-19 Vaccine: Hope and reality

**DOI:** 10.4314/ahs.v20i4.3

**Published:** 2020-12

**Authors:** Mohamud Sheek-Hussein, Fikri M Abu-Zidan

**Affiliations:** 1 Institute of Public Health, College of Medicine and Health Sciences, UAE University, Al-Ain, United Arab Emirates; 2 Department of Surgery, College of Medicine and Health Sciences, UAE University, Al-Ain, United Arab Emirates

## Introduction

The novel COVID-19 pandemic started at the Chinese province of Hubei in December 2019 after which the WHO declared it to be a public global health emergency.^1^ The COVID-19 virus has a zoonotic source with similarities to other highly infectious zoonotic coronaviruses. COVID-19 infection is highly contagious and continues to spread in both developed and developing countries.^2^ It has caused more than fifty million infected cases and more than one million two hundred fifty thousand deaths worldwide.^3^ Recently, the World Health Organization (WHO) and United Nations Children's Fund (UNICEF) reported an alarming decline in childhood immunization around the world because of the disruption caused by the COVID-19 pandemic.^4^ Immunization significantly reduced disability and death related to vaccine-preventable diseases worldwide.^5^ Delivering essential immunization is important to protect the health, well-being, and economy of a community. Until now, COVID-19 disease does not have effective antiviral drugs nor a fully licensed approved vaccine. Implementing public health measures that aim to prevent the transmission of COVID-19 such as wearing a facemask, social distancing and frequent hand washing are a real challenge.^3^ COVID-19 severity is increased in those having comorbidities and those having strong immunological reactions. While it is important to study treatment options for COVID-19 disease, an effective vaccine against COVID-19 is more rewarding because it will stop its spread. There is an urgent need for safe, effective, and long-standing high immunogenicity COVID-19 vaccines.

## Lessons from history

Development of a vaccine has always been difficult. It requires extensive experimental and clinical testing before being approved by the regulatory bodies. This may take years before approval.^5^, ^6^ During this long period, it will be difficult for public health measures alone to eliminate the virus. Just to highlight this point, in 1796 Edward Jenner inoculated a 13 year-old-boy with vaccinia virus (cowpox) and demonstrated immunity to smallpox. In 1798, the first smallpox vaccine was developed. Systematic mass immunization resulted in global eradication of smallpox only in 1979.^7^, ^8^ There have been several attempts to develop vaccines against recent emerging viral infections such as the Zika virus, Ebola, SARS and MERS-CoV. These attempts were disappointing. Difficulties encountered were the rapid genetic mutation of the pathogens and finding an appropriate animal model for testing. Similarly, the immune mechanisms against the three most devastating global diseases, malaria, tuberculosis and HIV are still poorly understood.^9^ We have also to stress that the development of a weak immunogenic vaccine could worsen the COVID-19 pandemic if authorities wrongly assume it causes a substantial reduction in risk.^10^

## One globe one health approach

The “one globe one health approach” is essential for the global fight against the COVID-19 pandemic. The scientific community and progress in finding a vaccine is affected by using COVID-19 as a political tool between different parties. This delays the world from reaching its similar global target. We must appreciate that mass global immunization is the only way to address the COVID-19 pandemic. Participation in COVID-19 immunization trials will be essential to reduce the impact of COVID-19 disease. The magnitude of participation will depend on the community attitude towards vaccination. Accordingly, vaccine hesitancy in a community must be defined. There is high acceptance for a COVID-19 vaccine in the USA^11^, ^12^, EU^13^, ^14^ and China^15^ but this has to be defined in other countries. The cost of giving the immunization in poor countries is a real concern because immunization should be given globally to quickly eliminate the disease and to reduce the chance of genetic mutations.

## Changing our strategy

It is a real challenge to develop vaccines against “complex” infections such COVID-19. Although the vaccine development seems difficult, the reward is huge. This requires innovative thinking. Our knowledge of biotechnology, immunology, genetics and virology is expanding. Furthermore, advancements in molecular engineering and platform technology which were applied in the vaccine development for tuberculosis, Zika, HIV, and Ebola will be tremendously useful in the development of a COVID19 vaccine.^16^ A successful vaccine should cause a proper humoral reaction usually through the organization of proteins from microbes. Therefore, designing effective vaccines requires a shift in our strategy by finding a vaccine that can produce a quick immune response. Vaccination response can be measured by antibody titers. Majority of those who are vaccinated may produce high titers of antibodies. Nevertheless, some may produce no or low levels of protective antibodies (Non or low responders) because of genetic predisposition, immunosuppression, or other disorders. Furthermore, the human body will retain its own memory on how to fight that diseases in the future. Nevertheless, after a while, immunity might decline. Therefore, booster doses may be needed to raise immunity to optimal levels.

## The way forward

We think that getting a COVID-19 vaccine, although it has multiple challenges, is feasible but may take time if it is going to be safe and effective. Currently, there are 45 candidate vaccines in clinical evaluation, and ten more in phases II and III clinical trials.^17^ Which of these rapid developed vaccines will be effective against COVID 19 is still unknown? Developing a vaccine needs proper understanding of the immune response against the COVID-19 virus.^18^ We have to acknowledge that this knowledge is still limited. An effective vaccine would generate a safe reaction producing antibodies to COVID-19 antigens.^19^ Antibody induced protection may be different from the normal defence against viruses because of the resistant naturef COVID-19. Antibodies solely directed towards the SARS-CoV spike (S) protein have been able to counteract the virus and halt infection.^20^ Studies in progress try to define whether the S protein or its receptor-binding domain (RBD) have comparable value as immunization targets.^21^

Finally, we should be optimistic. The prospect of effective vaccine may be on the horizon. An ongoing clinical trial gave very promising results. Interim analysis of a study on a new vaccine which is given in two doses, showed that it was more than 90% effective at preventing COVID-19 infection. The vaccine which is a virus messenger RNA enters the human cells and stimulates the production of the coronavirus spike proteins which are the target for the human immune system. Whether this vaccine will give long term immunity or whether it can be used in different patient populations is still unknown.^22^
